# A Promising Combination: PACAP and PARP Inhibitor Have Therapeutic Potential in Models of Diabetic and Hypertensive Retinopathies

**DOI:** 10.3390/cells10123470

**Published:** 2021-12-09

**Authors:** Etelka Pöstyéni, Krisztina Szabadfi, György Sétáló, Robert Gabriel

**Affiliations:** 1 Department of Experimental Zoology and Neurobiology, University of Pécs, 7624 Pécs, Hungary; etelka91@gamma.ttk.pte.hu (E.P.); kriszta.szabadfi@gmail.com (K.S.); 2 Department of Medical Biology, Medical School, University of Pécs, 7624 Pécs, Hungary; gyorgy.setalo.jr@aok.pte.hu

**Keywords:** PACAP, olaparib, retina, hypertensive retinopathy, diabetic retinopathy, immunohistochemistry, morphometric analysis

## Abstract

Diabetes and hypertension are complex pathologies with increasing prevalence nowadays. Their interconnected pathways are frequently manifested in retinopathies. Severe retinal consequences and their tight connections as well as their possible treatments are particularly important to retinal research. In the present, work we induced diabetes with streptozotocin in spontaneously hypertensive rats and treated them either with PACAP or olaparib and alternatively with both agents. Morphological and immunohistochemical analyses were carried out to describe cell-specific changes during pathologies and after different treatments. Diabetes and hypertension caused massive structural and cellular changes especially when they were elicited together. Hypertension was crucial in the formation of ONL and OPL damage while diabetes caused significant differences in retinal thickness, OPL thickness and in the cell number of the GCL. In diabetes, double neuroprotective treatment ameliorated changes of calbindin-positive cells, rod bipolar cells and dopaminergic amacrine cells. Double treatment was curative in hypertensive diabetic rat retinas, especially in the case of rod bipolar and parvalbumin-positive cells compared to untreated or single-treated retinas. Our results highlighted the promising therapeutic benefits of olaparib and PACAP in these severe metabolic retinal disorders.

## 1. Introduction

Diabetes mellitus and hypertension are two common, chronic, multifactorial diseases with increasing prevalence and socioeconomic complications worldwide. They both tend to appear with increased frequency as the aging process advances and usually coexist in clinical practice, with elevated prevalence for hypertension in diabetic patients. Moreover, blood pressure control has been proven to reduce the risk factor of diabetes-related complications while high blood pressure seems to increase disease progression [1,2,3,4,5]. Additionally, in a cardiovascular risk study, Cheung and his colleagues described that 58% of the diabetic patients had elevated blood pressure while 56% of hypertensive patients had dysglycemia [6]. The considerable overlap in etiology and the many interacting pathomechanisms (oxidative stress, immune cell activation, inflammation, etc.) exhibited by both these diseases provide a basis for metabolic, micro- and macrovascular consequences that have severe influential effects on the retina, the most metabolically active tissue of the human body [7,8,9].

To make matters worse, diabetic retinopathy (DR) is a major well-known cause for blindness with an increasing tendency in the working population [10,11]. It affects nearly 80% of patients with diabetes after 10 years of disease progression, increasing to 90% after 20 years [12,13]. DR can also lead to short-term alterations in the physiological function and structure of the retina [14], while hypertension affects the retinal information processing mechanisms on a long-term basis. Because of that, tight control of blood pressure is an essential factor in preventing severe complications [15,16].

Selecting an appropriate therapy is usually a complicated issue, and sometimes a combination of different therapies proves to be the best possibility to ameliorate symptoms [17]. Keeping that in mind, the current study was carried out to evaluate the potential therapeutic effects of pituitary adenylate cyclase activating polypeptide (PACAP) and poly (ADP-ribose) polymerase (PARP) inhibitor in the treatment of these systemic diseases in three different models, including spontaneously hypertensive rats (SHR), streptozotocin (STZ)-induced diabetic rats and rats with both pathologies (SHR + STZ).

According to earlier work, PACAP attenuates impairments originating from metabolic retinal disorders both at the structural and functional level [18], while PARP inhibition plays a protective role against ischemic [19] and hypertensive [20] complications. In the induced diabetes model, PACAP promotes neuronal survival during the course of diabetic retinopathy [21]. On the other hand, PARP-1 inhibition has also been proven capable of ameliorating neuropathy in diabetes [22]. So far, plenty of beneficial effects of both agents have been investigated separately in diabetic conditions, but the question of whether (and how) PACAP and PARP inhibition could work together against the complex pathomechanisms of hypertension and diabetes in the retina remains unanswered.

The main purpose of this study was to provide evidence that cellular protective effects exist and are available against the adverse effects of the examined common metabolic diseases in the retina. With morphological and immunochemical approaches, we demonstrate that these potentially promising therapeutic agents possess enhanced protective abilities not only by themselves but also when applied simultaneously in our experimental model.

## 2. Materials and Methods

### 2.1. Animals and Treatments

All procedures abided by the ethical permission approved by the University of Pecs (BA02/2000–15024/2011) and followed the ARVO guidelines. Spontaneously hypertensive rats (with minimum blood pressure 140/90 mmHg) were obtained from a commercial source (Charles River Laboratories, Veszprém, Hungary), (*n* = 45) were 8 weeks old and were housed at the Animal House of the University of Pécs. All animals were kept under identical conditions: room temperature varied between 19 °C and 23 °C, room humidity was set between 40% and 70% and a minimum of 15 air changes per hour were provided. The light cycle was controlled, and the light hours were timed between 7 a.m. and 7 p.m. After 3 weeks of acclimatization, diabetes was induced by the injection of streptozotocin (70 mg/kg) (Sigma, Hungary) into the penile veins. The glucose concentration of the blood was measured before the induction and afterwards (Glucotrend Accu-Check System, Roche, Hungary) (Table S1). If the blood glucose levels were higher than 15 mmol/L, they were classified as diabetic. We excluded those animals from further examinations who recovered after the injection and showed normal blood glucose levels in the follow-up period. Most animals showed signs of severe diabetes with typical symptoms (polydipsia, polyuria, excessive weight loss). After 1 week of induced diabetes, they received PACAP (100 pmol/5 µL saline solution; 20 µM) and olaparib (PARP inhibitor) treatments. PACAP was injected into the vitreous body of the right eye with a Hamilton syringe three times (once a week) under anesthesia, the same volume of saline was injected into the left eye, while olaparib was applied through their drinking water (estimated 4 mg/kg daily). One day after the 3rd PACAP treatment, animals were sacrificed with an overdose of anesthetic (Table S1b).

### 2.2. Histology

Animals were anaesthetized with Isofluorane (Isoba**^®^** vet, USA) and then sacrificed by decapitation. After both eyes were removed, the following histological procedures were performed: the eyes were fixed in 4% paraformaldehyde (PFA; Merck, Hungary; dissolved in 0.1 M phosphate buffer (PB; Spektrum3D, Hungary), the eyecups were dissected and then these were embedded in epoxy resin (Durcupan ACM resin; Sigma-Aldrich, Hungary) as described in previous study [18]. The retinal sections were cut at 2 μm, and stained with toluidine blue (Sigma-Aldrich, Hungary) and studied with a Nikon Eclipse 80i microscope. We have taken the measurements with the SPOT Basic program. Central retinal areas within 1 to 2 mm from the optic disc were used for measurements (*n* = 5–7 measurements from one tissue block). We measured the cross-section of the retina from the outer limiting membrane (OLM) to inner limiting membrane (ILM) to determine retinal thickness and the width of each individual retinal layer (ONL—outer nuclear layer; OPL—outer plexiform layer; INL-inner nuclear layer; IPL—inner plexiform layer) for further analysis. Cell numbers in the ganglion cell layer (GCL) were measured for lengths of 100 μm from sections. Statistical comparisons were made using the one-way ANOVA test followed by Tukey-B post hoc analysis. Data were presented as mean ± SEM (GraphPad Prism 5.0).

### 2.3. Immunhistochemistry

Dissection of the eyes was carried out in ice-cold phosphate buffer with saline (PBS) and fixed at room temperature in PFA (4%). Then, tissues were washed in PBS and cryoprotected in 15% and 30% sucrose at 4 °C. Retinas were embedded in tissue-freezing medium (Shandon Cryomatrix, USA) and cut with a cryostat (Leica, Germany) at 10 μm. Peanut agglutinin (PNA) conjugated with fluorescein isothiocyanate (FITC) and primary antibodies (anti-calbindin; anti-PKCα; anti-TH; anti-parvalbumin; anti-calretinin; anti-vGlut1; anti-GFAP; anti-GS) were used at room temperature overnight (Table S2). Next day, the sections were incubated with the corresponding secondary fluorescent antibodies (anti-mouse IgG conjugated with Alexa Fluor “488”; anti-rabbit IgG conjugated with Alexa Fluor “568”; anti-rabbit IgG conjugated with Alexa Fluor “488”; Table S1) for 2 h, in the dark, at room temperature and then cover slipped with Fluoromount-G (Southern Biotech, USA). Photos were taken with Fluoview FV-1000 Laser Confocal Scanning Microscope (Olympus, Japan).

## 3. Results

### 3.1. Morphological and Morphometric Analysis

**Figure 1:**
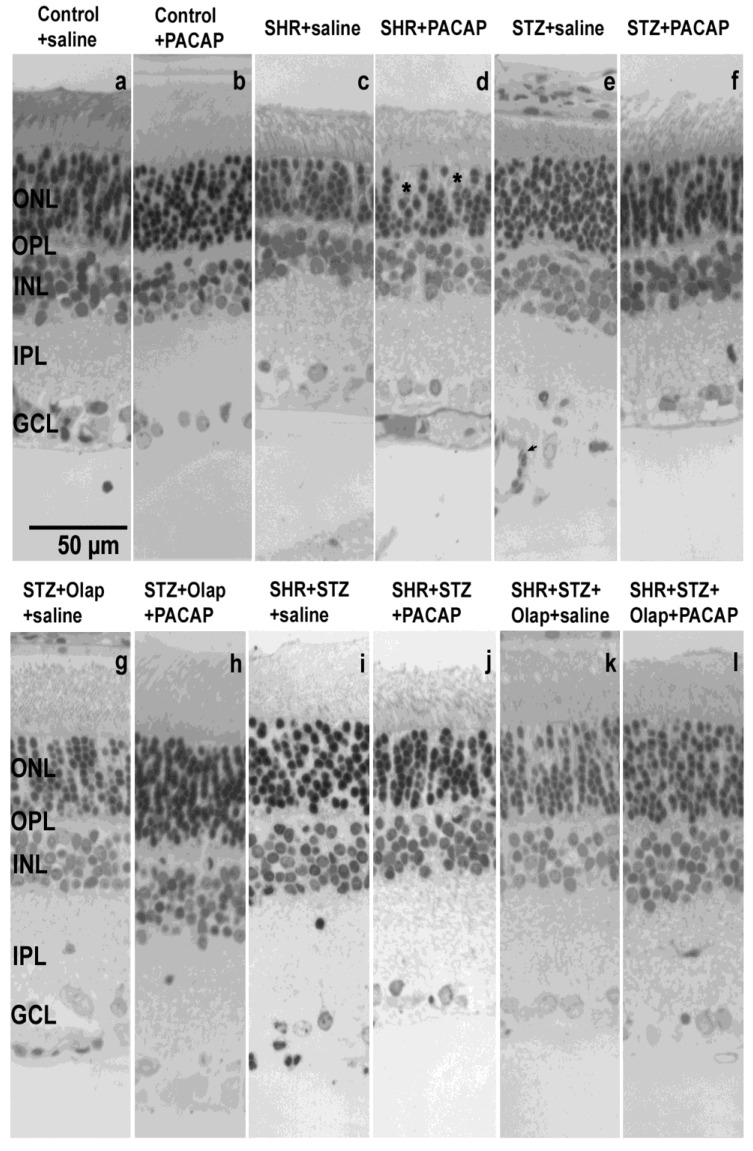
Representative sections of rat retinas in different conditions: (**a**) untreated control retina; (**b**) PACAP-treated control retina; (**c**) untreated hypertensive retina; (**d**) PACAP-treated hypertensive retina; (**e**) untreated diabetic retina; (**f**) PACAP-treated diabetic retina; (**g**) PARP inhibitor (Olap)–treated diabetic retina; (**h**) PACAP- and PARP inhibitor (Olap)–treated diabetic retina; (**i**) untreated hypertensive diabetic retina (**j**) PACAP-treated hypertensive diabetic retina; (**k**) PARP-inhibitor-treated hypertensive diabetic retina; (**l**) PACAP- and PARP inhibitor (Olap)–treated hypertensive diabetic retina. OLM—outer limiting membrane; ONL—outer nuclear layer; OPL—outer plexiform layer; INL—inner nuclear layer; IPL—inner plexiform layer; GCL—ganglion cell layer; ILM—inner limiting membrane. Arrows indicate capillaries, stars indicate signs of cellular damage.

The structural changes present in the retina (Figure 1) were revealed through morphometric analyses. The layered structure of the retina (ONL; OPL; INL; IPL; GCL) was recognizable in each case with typical retinal cells present. The presence of retinal diseases or effect of different treatments resulted in an alteration in the appearance of retinal layers between groups listed below. Besides the apparent structural changes in layer integrity, the change of cellular density and thickness of different layers, we have noticed signs of cellular damage (pyknotic nuclei, lack of clearly defined cytoplasm) in the ONL and capillary invasion with large lumen capillaries present primarily in the IPL and GCL.

**Figure 2:**
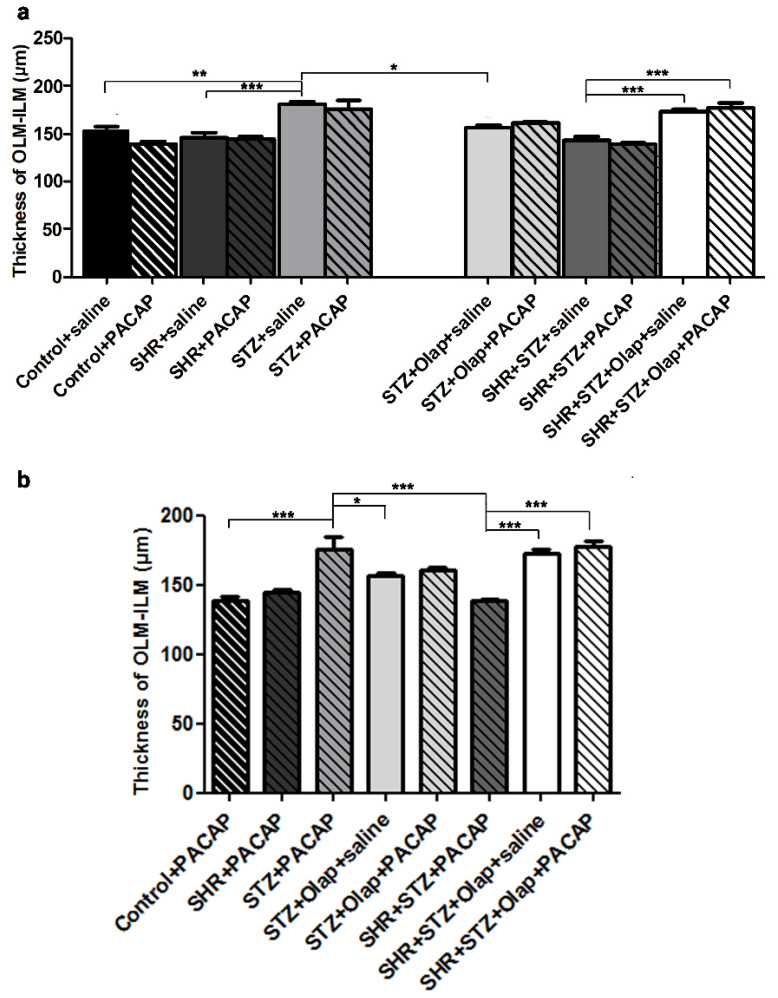
Thickness of OLM–ILM in the PACAP- or*/*and PARP inhibitor (Olap)–treated and untreated groups of hypertensive and diabetic rat retinas (**a**,**b**). Data are presented as mean ± SEM, where *** *p* < 0.001 and ** *p* < 0.01, * *p* < 0.05.

The treatments turned out to be helpful in preserving the ONL cells, especially in the case of the hypertensive diabetic groups (Figure 1). The retinal thickness changed significantly in different pathologies and after combined treatments (Figure 2). The method of measurement is described in the Material and Methods section in detail.

**Figure 3:**
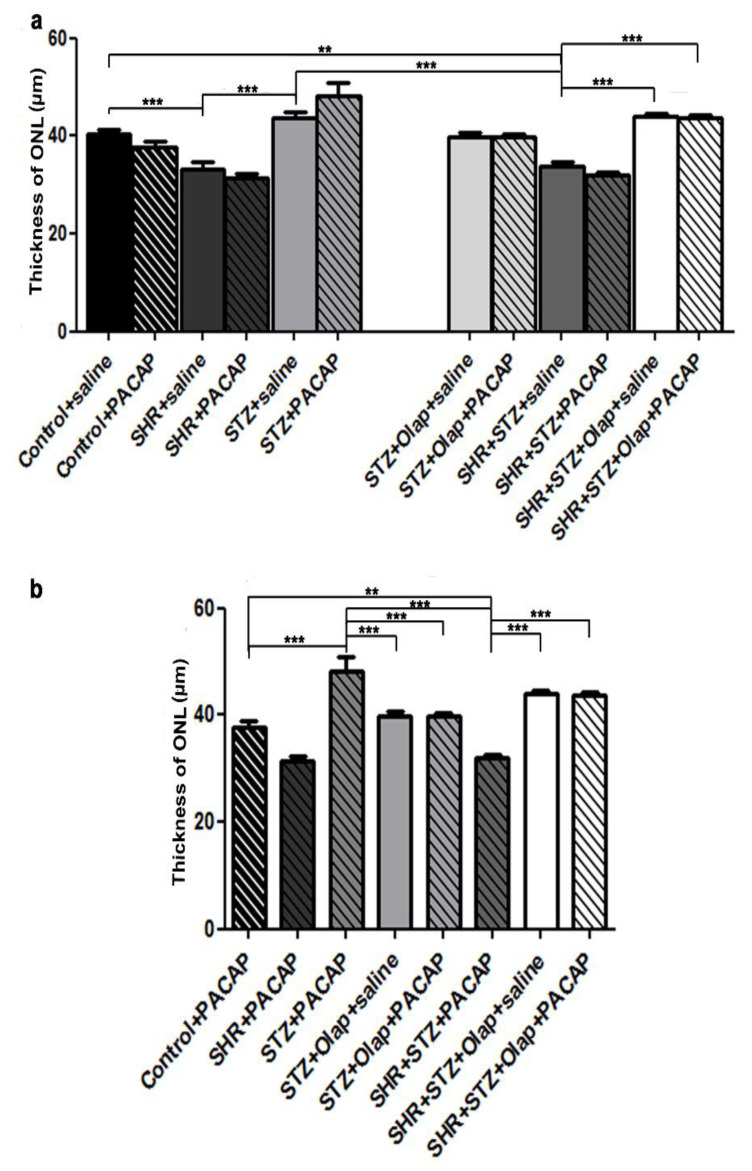
Thickness of ONL in the PACAP- or/and PARP inhibitor (Olap)–treated and untreated groups of hypertensive and diabetic rat retinas (**a**,**b**). Data are presented as mean ± SEM, where *** *p* < 0.001 and ** *p* < 0.01.

**Figure 4:**
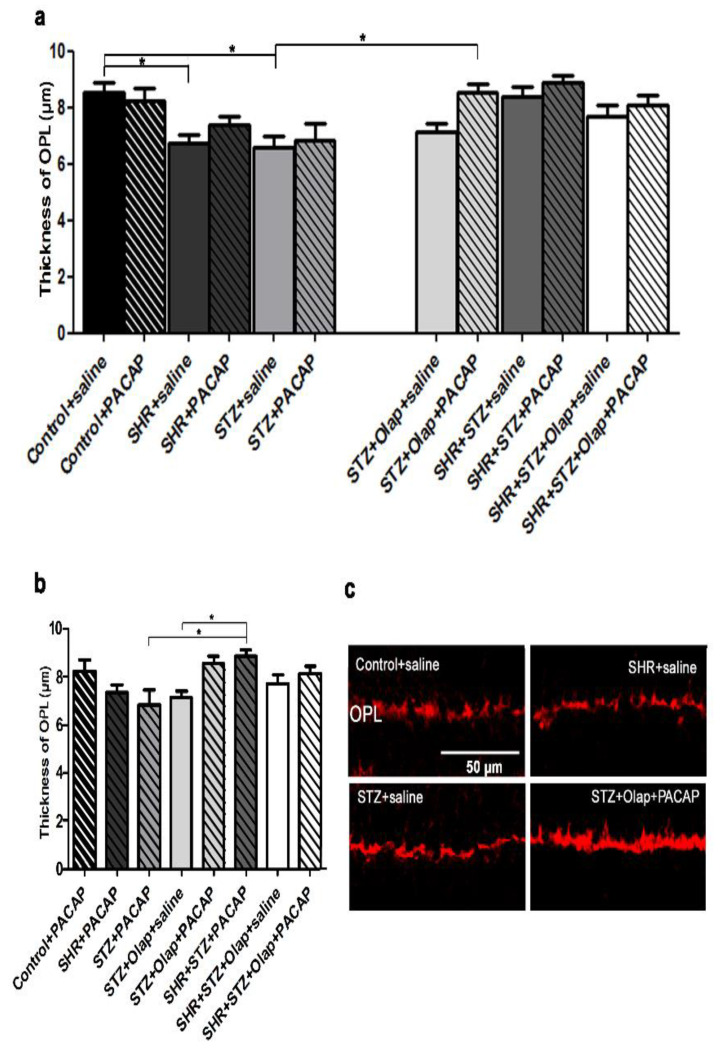
Thickness of OPL in the PACAP- or/and PARP inhibitor (Olap)–treated and untreated groups of hypertensive and diabetic rat retinas (**a**,**b**). Data are presented as mean ± SEM, where * *p* < 0.05. Representative images of OPL thickness, stained for vGlut1 (**c**). Scale bar: 50 µm.

**Figure 5:**
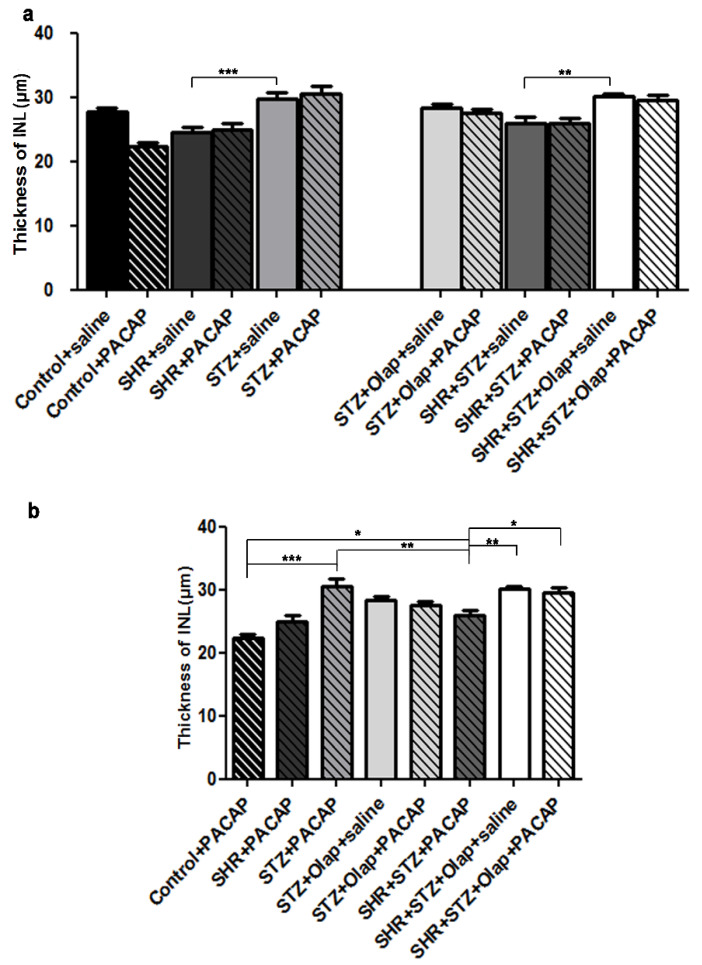
Thickness of INL in the PACAP- or/and PARP inhibitor (Olap)–treated and untreated groups of hypertensive and diabetic rat retinas (**a**,**b**). Data are presented as mean ± SEM, where *** *p* < 0.001, ** *p* < 0.01, * *p* < 0.05.

**Figure 6:**
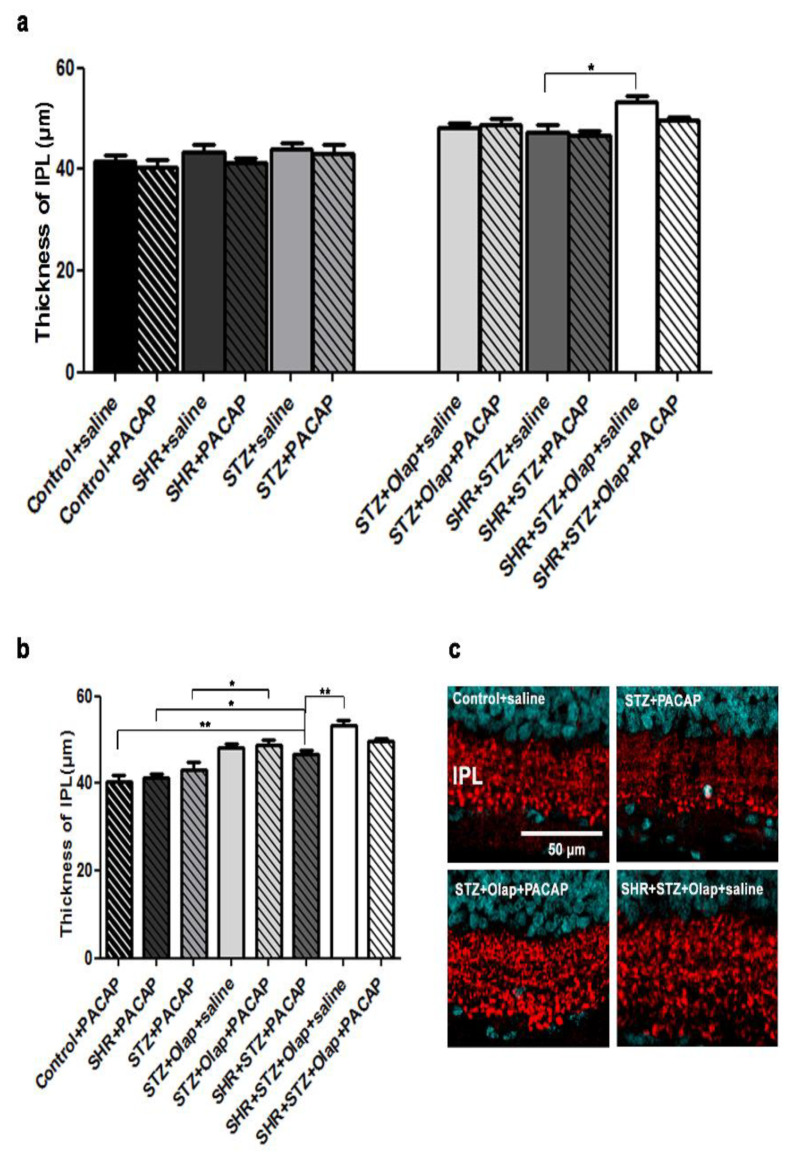
Thickness of IPL in the PACAP- or/and PARP inhibitor (Olap)–treated and untreated groups of hypertensive and diabetic rat retinas (**a**,**b**). Data are presented as mean ± SEM, where ** *p* < 0.01, * *p* < 0.05. Representative images of the IPL thickness, stained for vGlut1 (**c**). Scale bar: 50 µm.

We have found significant differences between the untreated control group and the untreated diabetic group, which also showed significant differences compared to the PARP-inhibitor-treated diabetic group and the untreated hypertensive group (Figure 2a). The OLM–ILM distance was significantly greater in the PACAP-treated diabetic group than that in the PACAP-treated control, the PARP-inhibitor-treated diabetic and the PACAP-treated hypertensive diabetic group. The PACAP-treated hypertensive diabetic group had significantly less OLM–ILM distance than the double treated and the PARP-inhibitor-treated hypertensive diabetic retinas (Figure 2b). The influence of PACAP and PARP-inhibition treatments were especially visible in the diabetic and hypertensive retinas both because of their effect on retinal thickness and due to the changes provoked in individual layers (Figure 3, Figure 4, Figure 5 and Figure 6).

The ONL thickness was significantly less in the untreated hypertensive group than in the untreated control and the untreated diabetic group (Figure 3).

In the untreated hypertensive diabetic group, the ONL thickness was significantly reduced compared to the untreated control or to the untreated hypertensive retinas. In addition, the untreated hypertensive diabetic group also was significantly different from the PARP-inhibitor-treated hypertensive diabetic group and the double treated hypertensive diabetic group (Figure 3a). The treatments turned out to be helpful in preserving the ONL cells, especially in the case of the hypertensive diabetic groups. The ONL thickness was significantly greater in the PACAP-treated diabetic retinas than in the PARP-inhibitor-treated diabetic retinas, the PACAP-treated hypertensive diabetic retinas or the double-treated diabetic retinas. The ONL thickness was significantly less in the PACAP treated hypertensive diabetic retinas than in the PARP-inhibitor-treated or double-treated hypertensive diabetic retinas (Figure 3b).

The thickness of the OPL changed most as a consequence of the presence of diabetes or hypertension (Figure 4).

The OPL thickness decreased significantly in the untreated hypertensive and in the diabetic group compared to the untreated control retinas. The OPL thickness was significantly greater in the double-treated diabetic group than in the untreated diabetic group (Figure 4a,c). The PACAP-treated hypertensive group was significantly different compared to the PARP-inhibitor-treated and the PACAP-treated diabetic group (Figure 4b). The double treatments were effective in the preservation of the OPL thickness, and PACAP treatment also had a positive effect compared to the untreated groups (Figure 4c).

The INL thickness was greater in the untreated diabetic group than in the untreated hypertensive group. Moreover, it is significantly increased in the PARP-inhibitor-treated hypertensive diabetic group compared to their untreated hypertensive diabetic control (Figure 5a).

In addition, the INL thickness was significantly less in the PACAP-treated hypertensive diabetic group than in the PACAP-treated diabetic group, the PARP-inhibitor-treated hypertensive diabetic group or the double-treated hypertensive diabetic group. The PACAP-treated diabetic group had a thicker INL than the PACAP-treated control, which was significantly different compared to the PACAP-treated hypertensive diabetic group (Figure 5b).

The thickness of IPL significantly increased in the PARP-inhibitor-treated hypertensive diabetic group compared to their untreated hypertensive diabetic control (Figure 6a). The PACAP-treated hypertensive diabetic group had significantly thicker IPL than the PACAP-treated control and the PACAP-treated hypertensive group. The PACAP-treated diabetic group had less IPL thickness compared to the double-treated diabetic group. Furthermore, the PARP-inhibitor-treated hypertensive diabetic group showed greater IPL thickness than the PACAP-treated hypertensive diabetic group (Figure 6b,c).

**Figure 7:**
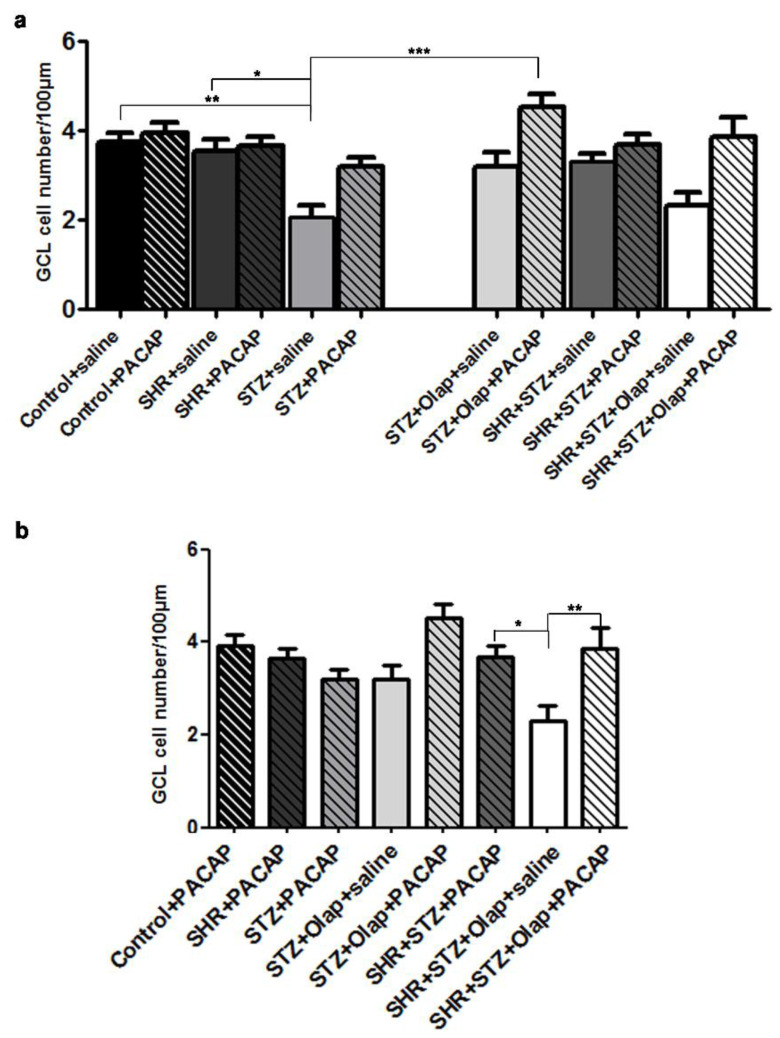
Number of cells in the ganglion cell layer in the PACAP- or/and PARP inhibitor (Olap)–treated and untreated groups of hypertensive (SHR) and diabetic (STZ) rat retinas (**a**,**b**). Cell numbers in ganglion cell layer were measured in retinal sections for lengths of 100 μm. Data are presented as mean ± SEM, where *** *p* < 0.001, ** *p* < 0.01, * *p* < 0.05.

The cell numbers of the GCL (Figure 7) were significantly less in the untreated diabetic retinas compared to the untreated hypertensive, the control retinas and the double-treated diabetic retinas (Figure 7a). Moreover, the double-treated hypertensive diabetic group had higher cells number in the GCL than the PARP-inhibitor-treated hypertensive diabetic group. To conclude, double treatment proved to be more effective against these pathologies compared to the single treatment or control.

### 3.2. Immunohistochemistry

To reveal the specific effects of the examined diseases or treatments at the cellular level, we conducted immunohistochemical analysis using different markers for identification (PKCα, TH, calbindin, calretinin, parvalbumin, GFAP, GS, PNA, vGlut1). Considering the extensiveness of the research data obtained from these experiments, we only present the results for those that showed remarkable deviation from our expectations and previously published results (PKCα, TH, calbindin, calretinin, parvalbumin, vGlut1). At the same time, all other supporting are available as Supplementary Materials (Figure S6—GS, Figure S7—GFAP; Figure S8—PNA; Figure S9—vGlut1).

**Figure 8:**
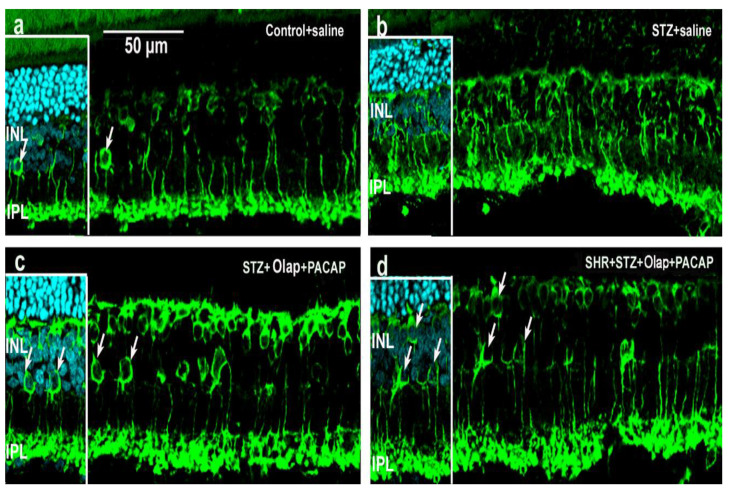
Representative retina sections stained with rod bipolar cell marker (PKCα) in different conditions. Inserted images include DAPI staining. (**a**) Untreated control retina; (**b**) untreated diabetic retina; (**c**) PARP inhibitor (Olap)– and PACAP-treated diabetic retina; (**d**) PARP inhibitor (Olap)– and PACAP-treated hypertensive diabetic retina. INL—inner nuclear layer, IPL—inner plexiform layer. Scale bar: 50 µm.

According to our results, the organization of the retina is clearly disrupted in diabetics (Figure 8b) compared to the control (Figure 8a). After PACAP and PARP inhibitor treatments (Figure 8c,d), an almost fully preserved regular structure is seen, with similar results for combined diabetic and hypertensive conditions. PKCα in rod bipolar cells showed lower intensity in the untreated diabetic (Figure 8b) retina than in control retinas (Figure 8a). In the double-treated retina, the labeling intensity increased and layers remained distinct (Figure 8c,d and Figure S1).

**Figure 9:**
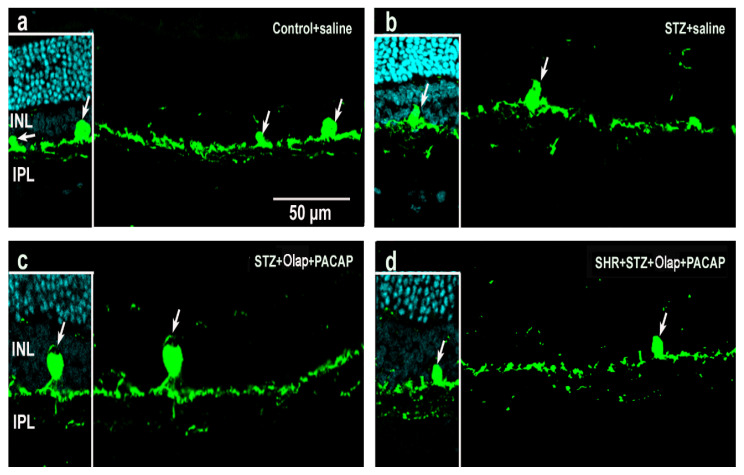
Representative retina sections stained with dopaminergic amacrine cell marker (TH) in different conditions. Inserted images include DAPI staining. (**a**) Untreated control retina; (**b**) untreated diabetic retina; (**c**) PARP inhibitor (Olap)– and PACAP-treated diabetic retina; (**d**) PARP inhibitor (Olap)– and PACAP-treated hypertensive diabetic retina. INL—inner nuclear layer, IPL—inner plexiform layer. Scale bar: 50 µm.

Tyrosine hydroxylase (TH) was localized in dopaminergic amacrine cells in the INL (Figure 9 and Figure S2). Labeled somata occurred with irregular shape in the diabetic group, and their dendritic branching showed lower labeling intensity (Figure 9b) compared to control. After double treatments were carried out, the shape of the cells remained regular in retinas from the diabetic group (Figure 9c). However, the deterioration of shape and branching of dopaminergic amacrine cells could not be prevented for the hypertensive diabetic group even with combined PACAP + PARP inhibitor treatment (Figure 9d).

**Figure 10:**
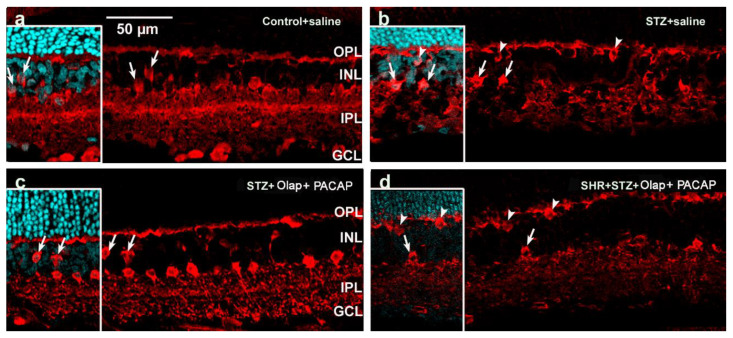
Calbindin immunoreactivity in retina sections under different conditions: (**a**) untreated control. Inserted images include DAPI staining. (**b**) Untreated diabetic retina; (**c**) PACAP- and PARP inhibitor (Olap)–treated diabetic retina; (**d**) PACAP- and PARP inhibitor (Olap)–treated hypertensive diabetic retina. OPL—outer plexiform layer, INL—inner nuclear layer, IPL—inner plexiform layer, GCL—ganglion cell layer. Scale bar: 50 µm.

Calbindin immunoreactivity (Figure 10 and Figure S3) occurred in control retinas mainly in horizontal cells and also in some ganglion and amacrine cells (Figure 10a). In diabetic retinas, the labeling of structures became disrupted (Figure 10b), but increasing calbindin immunoreactivity was observed after PACAP + PARP inhibitor treatments, and the retina structure in these retinas was also well preserved (Figure 10c). In hypertensive diabetic retinas, the organization of the retina remained damaged even after the combined treatment (Figure 10d), indicating that hypertension is critical in the development of this pathology.

**Figure 11:**
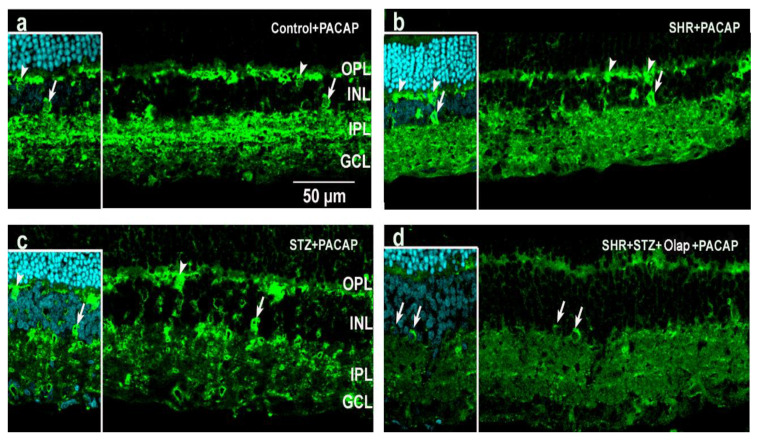
Calretinin immunoreactivity in retinal sections under different conditions: (**a**) PACAP-treated control retina. Inserted images include DAPI staining. (**b**) PACAP-treated hypertensive retina; (**c**) PACAP-treated diabetic retina; (**d**) PACAP- and PARP inhibitor (Olap)–treated hypertensive diabetic retina. OPL—outer plexiform layer, INL—inner nuclear layer, IPL—inner plexiform layer, GCL—ganglion cell layer. Scale bar: 50 µm.

Calretinin was detected in the amacrine and ganglion cells (Figure 11a and Figure S4) and showed alterations in hypertensive (Figure 11b) and hypertensive diabetic retinas (Figure 11d). The labeling of the three sublaminas of the IPL became diffuse and disappeared in these pathologies, and the treatments were not able to preserve and restore the original calretinin distribution (Figure 11c,d). Hypertension seems to be a critical factor in the development of this pathology.

**Figure 12:**
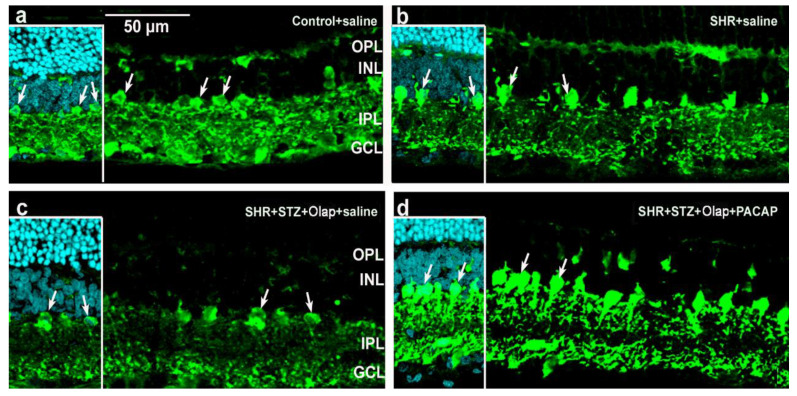
Parvalbumin immunoreactivity in retinal section with different conditions. Inserted images include DAPI staining. (**a**) Untreated control; (**b**) untreated hypertensive retina; (**c**) PARP inhibitor (Olap)–treated hypertensive diabetic retina; (**d**) PARP- and PACAP inhibitor (Olap)–treated hypertensive diabetic retina. OPL—outer plexiform layer, INL—inner nuclear layer, IPL—inner plexiform layer, GCL—ganglion cell layer. Scale bar: 50 µm.

Parvalbumin was detected in some amacrine cells and ganglion cells (Figure 12 and Figure S5). The intensity of the labeling increased in the double-treated hypertensive diabetic groups (Figure 12d) compared to the PARP-treated hypertensive diabetic groups (Figure 12c). The PACAP treatment had strong protective effects on the parvalbumin-positive cells in these pathologies.

Glutamine synthase (GS) levels increased in the retina in our experiments during diabetes (Figure S6), while levels of glial fibrillary acidic protein (GFAP) also followed this trend (Figure S7). In the combined presence of both diabetes and hypertension, the double treatment did not influence the expression of these proteins strongly, indicating non-specific metabolic stress. The double treatments have exhibited protective effects toward the PNA-labeled photoreceptor cells against different pathologies (Figure S8), which is in agreement with our previous observations [18]. The vesicular glutamate transporter 1 (vGlut1) level did not change remarkably, only the double-treated diabetic retinas showed slight increase in labeling (Figure S9). At the same time, we used this marker to demonstrate the thickness changes of the plexiform layers as shown earlier (Figure 4c and Figure 6c).

## 4. Discussion

Structural changes and cell death are two main consequences of retinal pathologies, which lead to altered cellular communication and functional decline. In the present work, we have described the effects of two promising therapeutic agents against impairments during the manifestation of the most common metabolic origins of pathologies in the retina: hypertension and diabetes. Besides the tight control of blood glucose level, a good control of hypertension is also an important step during the clinical management of DR, because they usually occur together in clinical practice and have a reciprocal connection. Among their cellular and molecular processes, we could find several common, connected impairment mechanisms, such as neurodegeneration, loss of synaptic connectivity, glial activation or the neurovascular unit dysfunction [18,19,23,24], which provide explanations for the tight relationship between them. Research into their pharmacological management have elucidated their complicated, interwoven processes and have initiated the need to develop new combined therapeutic approaches.

Increased level of cell death, impairment of survival pathways, inflammation and nitrative stress were described as destructive consequences of hypertensive diabetes in the rat retina. These processes were more serious in the hypertensive diabetic group compared to groups only with hypertension or with diabetes. At the molecular level, the increase of JNK phosphorylation, glial reactivity, VEGF, ICAM, lipid peroxide, nitrotyrosine, NFkB, p65 and TNFα levels are also well described [25,26]. Dozens of studies described the role of PARP as nuclear enzyme in DNA-damage-induced cell death processes and its increased activity connected to the manifestation of several neurodegenerative pathologies. There are few PARP inhibitors that have effectively proved their therapeutic effects against different pathologies and confirmed their protective effect also in retinal disorders. Pharmacological inhibition of PARP offers protection during photoreceptor degeneration in retinitis pigmentosa [27,28], hypoxia-induced ischemic injury [19], hyperfusion-induced neurodegeneration [29] and diabetic retinal complication [30,31] in the retina. On the other hand, PACAP, as an endogenous neuropeptide, proved its neuroprotective roles in neurodegenerative disorders[32,33,34] and also in retinal pathologies such as retinal ischemia [35], excitotoxic retinal injury [36] or in DR [18,21,37,38,39,40]. The protective roles of PARP inhibitors and PACAP treatment have been proved to be connected with these altered molecular pathways in the background of cell death and survival. Absence or inhibition of PARP decreased inflammation, suppressed NFKB p65 activation, attenuated MAPK activation, reduced nitrotyrosine formation and induced AKT phosphorylation. On the other hand, PACAP treatment reduced activated caspase levels, attenuated p38MAPK protein levels and phosphorylation of pro-apoptotic p38MAPK and increased Akt phosphorylation, ERK1, PKC and Bcl-2 protein levels in diabetic retinas [21].

Our results showed that both hypertension and DR caused massive structural and cellular changes in the retina especially when they were elicited together. The different degrees of changes in rat retina were previously described during induced diabetes [18,41,42,43] in hypertension [44,45] and in diabetic hypertensive rats [46,47]. Combined treatment with PACAP and PARP inhibitor could attenuate this severe alteration in different ways.

As we have shown in this study, the retinal thickness was larger in the diabetic retinas than in the untreated hypertensive group. PARP inhibitor treatment in the diabetic group and the double treatment in the hypertensive diabetic group retinas could attenuate these OLM–ILM distance alterations. The alteration of retinal thickness was described previously in induced diabetes [18,48], where PACAP treatment could attenuate this [18]; moreover, olaparib treatment also rescued the retinal tissue in hypoxia/reoxygenation (H/R) [19]. The effects of PACAP and PARP inhibitors on the attenuation of retinal layer reduction has also been described in a model of retinal hypoperfusion [29,49]. The double treatment was also curative against the OPL reduction in diabetes and against ONL reduction in hypertensive diabetic retinas. In a previous investigation, we described that PACAP attenuates changes of the OPL in induced diabetes [18], and PARP also proved to be protective against this change in H/R retinal injury [19]. The combined treatment with PARP inhibitor and PACAP was mainly helpful against the reduction of the cell number in the GCL in diabetic retinas compared to single treatment. In previous investigations, PARP inhibitors and PACAP helped in the prevention of ganglion cell number decline during optic nerve transection [50,51]. Moreover, olaparib was protective in H/R-induced retinal injury [19], PACAP in induced diabetes [18] and ischemia [52] by promoting ganglion cell survival.

The effectiveness of double treatment was also confirmed by our immunohistochemistry results. The double treatment increased the PKC alpha immunoreactivity in rod bipolar cells, and TH reactivity in dopaminergic amacrine cells in SHR + STZ rats; moreover, the double treatment was also more effective at increasing the calcium binding proteins’ expressions. PACAP treatment could ameliorate the decrease of TH-positive cells and the bipolar cells [18]. Calbindin and calretinin expression were the worst in the diabetic group, the combined treatment had a stronger curative effect than the PACAP treatment. Moreover, hypertension was critical in their altered expression. Parvalbumin expression showed another trend. Diabetes really disrupted its expression, but PACAP treatment could moderate it, and double treatment with olaparib further enhanced protection.

In summary, we introduced a new combined pharmacological tool in the treatment of retinal consequences of hypertension and induced diabetes in animal models. Being aware of the increasing prevalence of these pathologies in the human population, there is a need to develop their clinical management. Our work highlighted how promising therapeutic benefits of PARP inhibitor and PACAP can be utilized in the management of these severe metabolic retinal disorders.

## Data Availability

The data presented in this study are available in Supplementary Materials.

## Supplementary Materials

The following are available online at https://www.mdpi.com/article/10.3390/cells10123470/s1, Table S1. (a) Final blood glucose level and initial and final body weight in different groups. (b) Timeline of the induction of diabetes, treatment of olaparib and PACAP. Table S2. Primary and secondary antibodies used in the immunohistochemical analysis (IHC), Figure S1. Representative retinal sections stained with rod bipolar cell markers (PKCα) in different conditions, Figure S2. Representative retinal sections stained with dopaminergic amacrine cell marker (TH) in different conditions, Figure S3. Calbindin immunoreactivity in retinal sections under different conditions, Figure S4. Calretinin immunoreactivity in retinal sections under different conditions, Figure S5. Parvalbumin immunoreactivity in retinal sections under different conditions, Figure S6. Representative retinal sections stained with GS in different conditions, Figure S7. Representative retinal sections stained with Müller glia cell marker (GFAP) in different conditions, Figure S8. Representative retinal sections stained with cone terminal marker (PNA) in different conditions, Figure S9. vGlut1 immunoreactivity in photoreceptor and bipolar cell terminals.
