# Supplementary material for: A Promising Combination: PACAP and PARP Inhibitor Have Therapeutic Potential in Models of Diabetic and Hypertensive Retinopathies
Source: Cells. 2021 Dec 9;10(12):3470. doi: 10.3390/cells10123470 (PMC8700737; doi:10.3390/cells10123470)
Supplement: Supplementary file 1 [file cells-10-03470-s001.zip › cells-1494268-supplementary.pdf]

cells

MDPI

*Article*

# A promising combination: PACAP and PARP inhibitor have therapeutic potential in models of diabetic and hypertensive retinopathies

**Etelka Pöstyéni [1], Krisztina Szabadfi [1], György Sétáló Jr.[2] and Robert Gabriel [1,*]**

[1] Department of Experimental Zoology and Neurobiology, University of Pécs, 7624 Pécs, Hungary; etelka91@gamma.ttk.pte.hu (E.P.); kriszta.szabadfi@gmail.com (K.SZ.) ; gabriel@ttk.pte.hu (R.G.)
[2] Department of Medical Biology, Medical School, University of Pécs, 7624 Pécs, Hungary; gyorgy.setalo.jr@aok.pte.hu (G.S.)
* Correspondence: gabriel@ttk.pte.hu

Table S1. (a) Final blood glucose level and initial and final body weight in different groups. Data are presented as mean ± SEM. (b) Timeline of the induction of diabetes, treatments of olaparib and PACAP.

**a**

| Groups | Glycemia (mmol/L) | Initial body weight (g) | Final body weight (g) |
|---|---|---|---|
| Control+PACAP | 7,05±0,27 | 339,8±34,32 | 342,3±36,97 |
| SHR+PACAP | 6,65±0,19 | 280,5±5,149 | 285,5±6,125 |
| STZ+PACAP | 25,85±0,75 | 396±1 | 359±8 |
| STZ+PACAP+Olap | 31,48±0,53 | 362,5±8,578 | 318,1±7,03 |
| SHR+STZ+PACAP | 30,45±1,65 | 288,5±8,5 | 217,5±12,5 |
| SHR+STZ+PACAP+Olap | 28,36±1,00 | 284,4±2,804 | 230±5,31 |

**b**

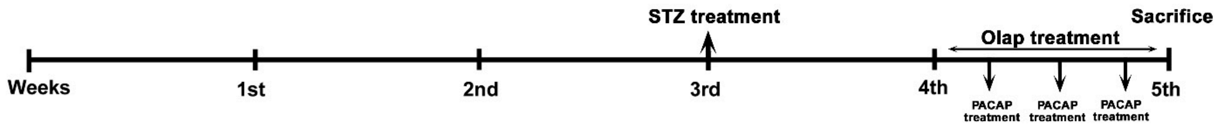

Table S2. Primary and secondary antibodies used in the immunohistochemical analysis (IHC).

| Catalogue number | Primary antibody | Raised in | Dilution | Manufacturer |
|---|---|---|---|---|
| FL1071 | PNA (FITC conjugated) | | 1:500 | VectorLab, UK |
| CB38 | anti-calbindin | rabbit | 1:1000 | Swant, USA |
| SC17804 | anti-PKC$\alpha$ | mouse | 1:200 | Santa-Cruz, USA |
| ab112 | anti-TH | rabbit | 1:1000 | Abcam, UK |
| PV27 | anti-parvalbumin | rabbit | 1:1000 | Swant, USA |
| CG1 | anti-calretinin | goat | 1:1000 | Swant, USA |
| ab77822 | anti-vGLUT1 | rabbit | 1:100 | Abcam, UK |
| G9269 | anti-GFAP | rabbit | 1:500 | Sigma, Hungary |
| PA5-35365 | anti-GS | rabbit | 1:1000 | Invitrogene, USA |
| **Catalogue number** | **Secondary antibody for IHC** | **Raised in** | **Dilution** | **Manufacturer** |
| A11001 | anti-mouse IgG conjugated with Alexa Fluor "488" | goat | 1:1000 | Life Technologies, USA |
| A11011 | anti-rabbit IgG conjugated with Alexa Fluor "568" | goat | 1:1000 | Life Technologies, USA |
| A11034 | anti-rabbit IgG conjugated with Alexa Fluor "488" | goat | 1:1000 | Life Technologies, USA |

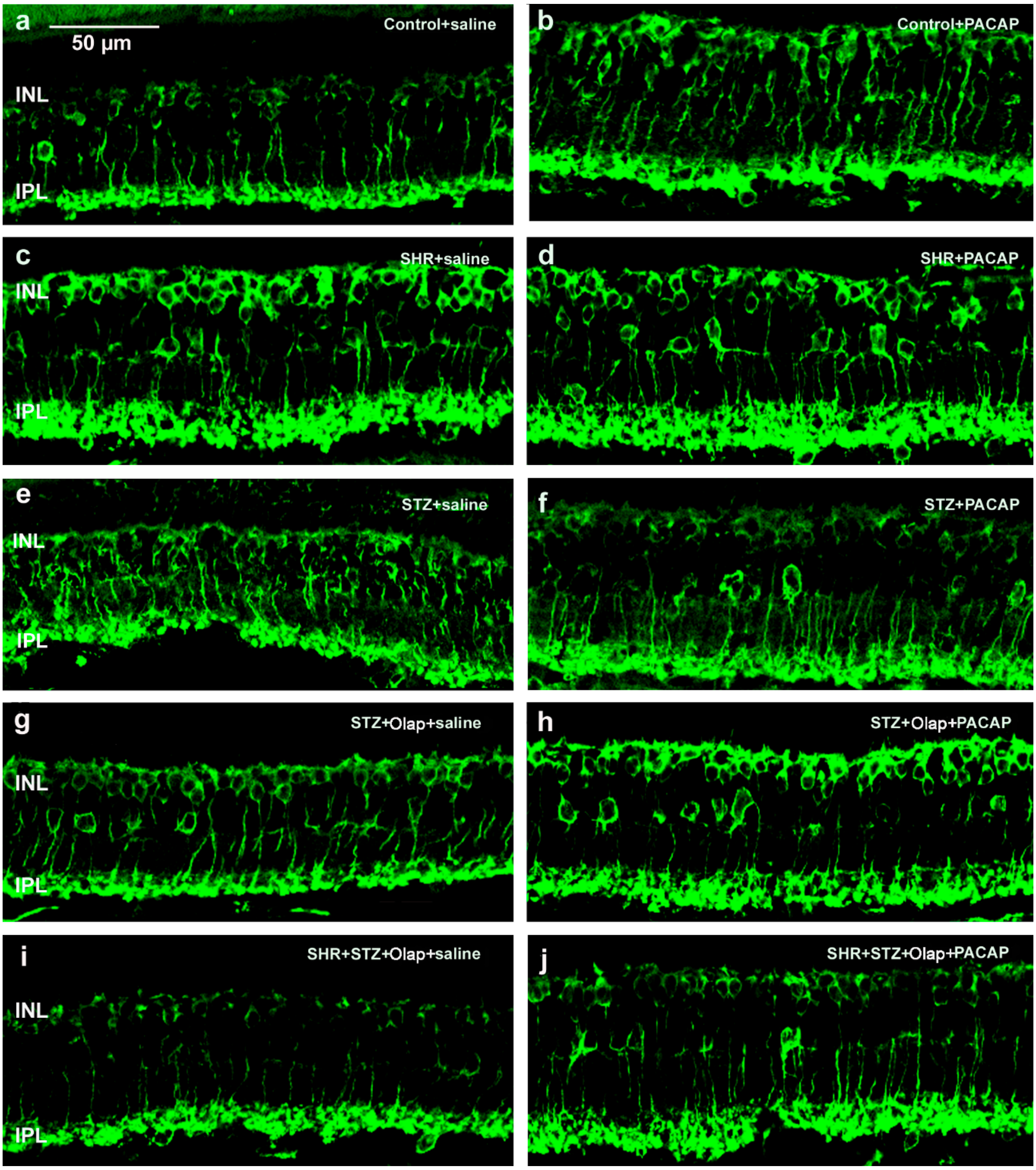


**Figure S1.** Representative retinal sections stained with rod bipolar cell markers (PKC $\alpha$) in different conditions: (**a**) untreated control retina; (**b**). PACAP treated control retina; (**c**) untreated hypertensive retina; (**d**) PACAP treated hypertensive retina, (**e**) untreated diabetic retina;(**f**) PACAP treated diabetic retina;(**g**) PARP treated diabetic retina;(**h**) PACAP and PARP inhibitor (Olap) treated diabetic retina;(**i**) PARP inhibitor (Olap) treated hypertensive diabetic retina;(**j**) PACAP and PARP treated hypertensive diabetic retina.. INL- inner nuclear layer, IPL- inner plexiform layer. Scale bar : 50μm.

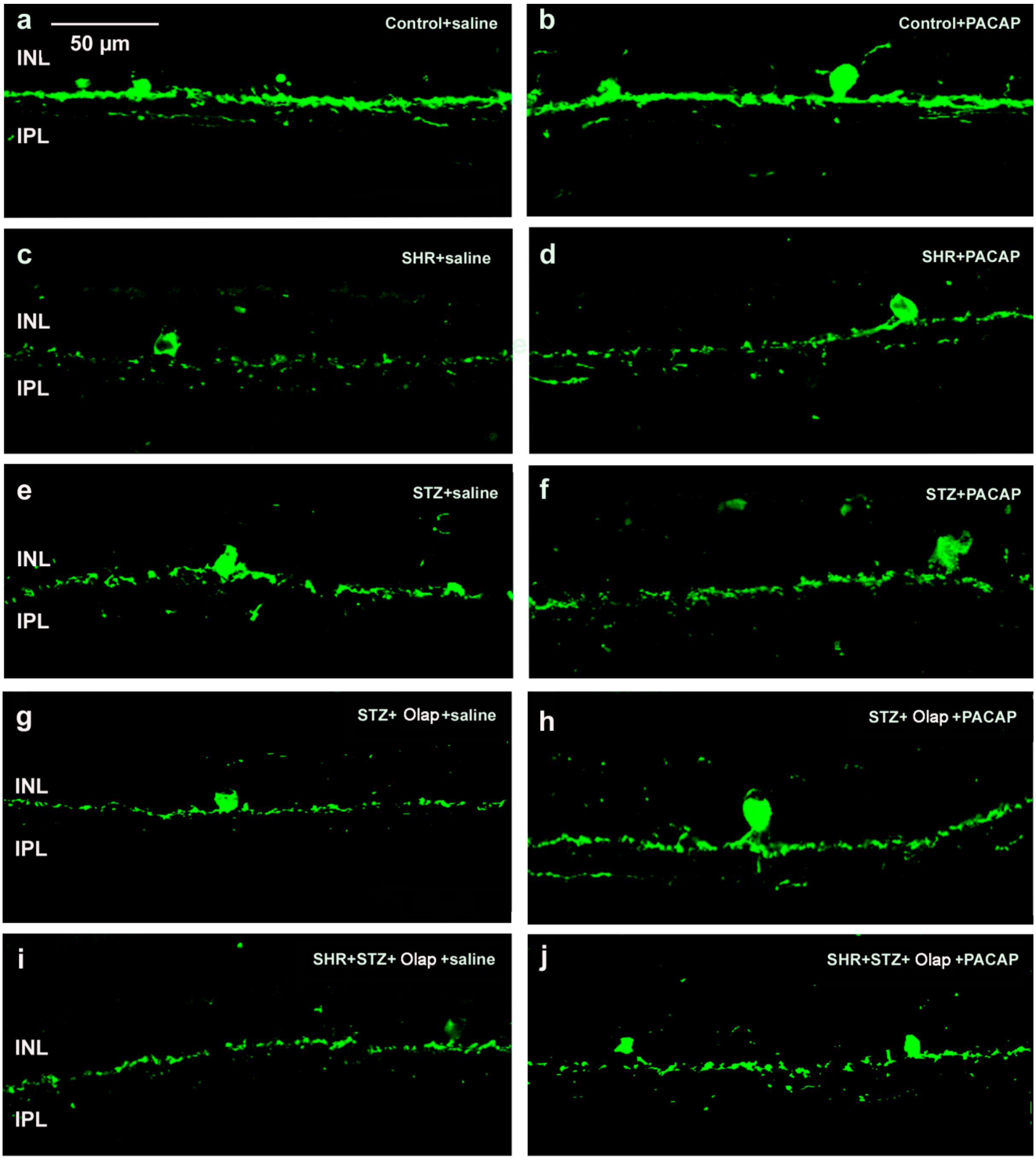


**Figure S2.** Representative retinal sections stained with dopaminergic amacrine cell marker (TH) in different conditions: (**a**) untreated control retina; (**b**) PACAP treated control retina; (**c**) untreated hypertensive retina; (**d**) PACAP treated hypertensive retina,(**e**) untreated diabetic retina; (**f**) PACAP treated diabetic retina; (**g**) PARP inhibitor (Olap) treated diabetic retina; (**h**) PACAP and PARP inhibitor (Olap) treated diabetic retina; (**i**) PARP inhibitor (Olap) treated hypertensive diabetic retina; (**j**) PACAP and PARP inhibitor (Olap) treated hypertensive diabetic retina. INL- inner nuclear layer, IPL- inner plexiform layer. Scale bar: 50µm.

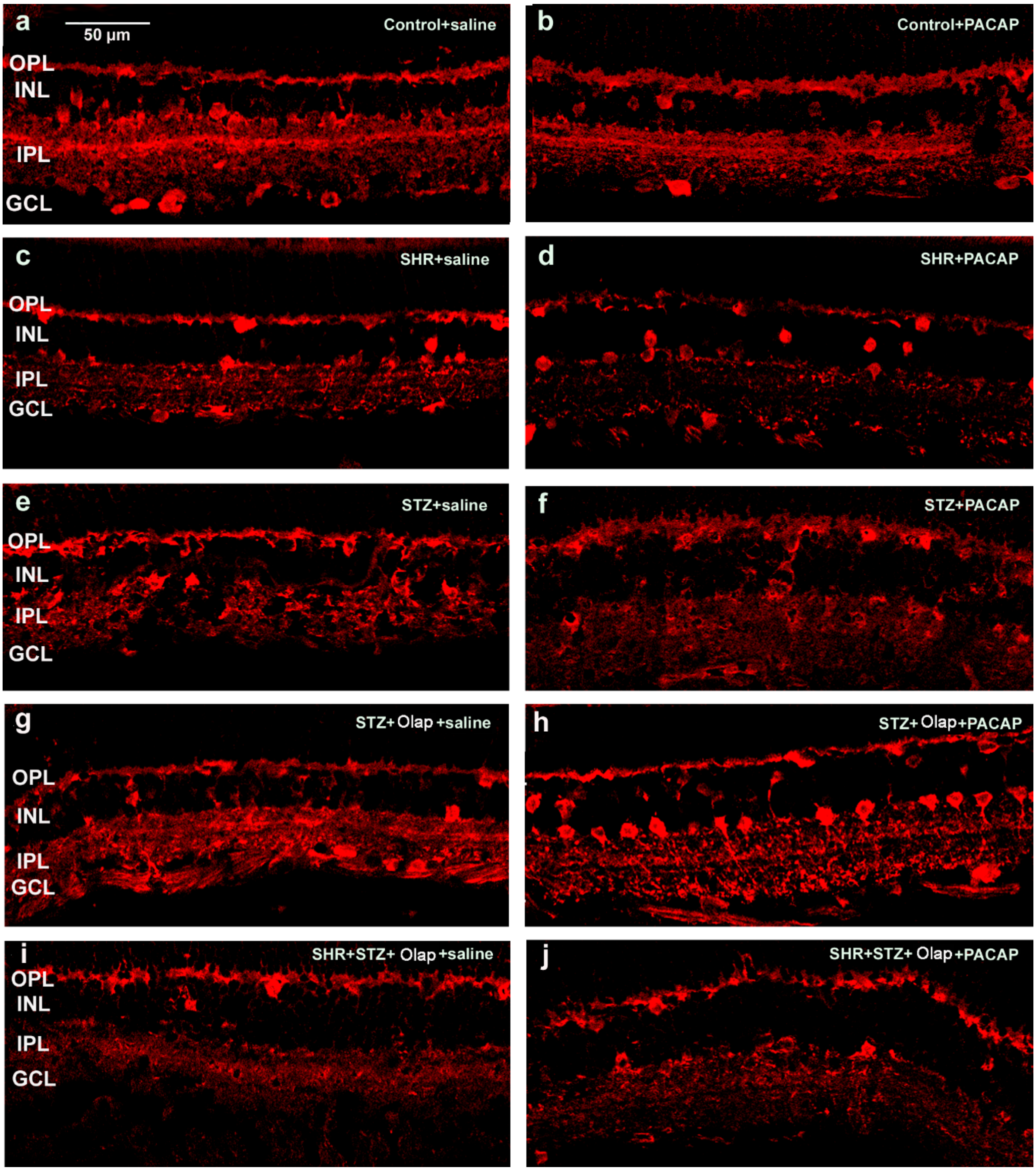


**Figure S3.** Calbindin immunoreactivity in retinal sections under different conditions: (**a**) untreated control retina; (**b**) PACAP treated control retina; (**c**) untreated hypertensive retina; (**d**) PACAP treated hypertensive retina; (**e**) untreated diabetic retina; (**f**) PACAP treated diabetic retina; (**g**) PARP inhibitor treated (Olap) diabetic retina; (**h**) PACAP and PARP inhibitor (Olap) treated diabetic retina; (**i**) PARP inhibitor (Olap) treated hypertensive diabetic retina; (**j**) PACAP and PARP inhibitor (Olap) treated hypertensive diabetic retina. Scale bar: 50µm. OPL- outer plexiform layer, INL- inner nuclear layer, IPL- inner plexiform layer, GCL-ganglion cell layer

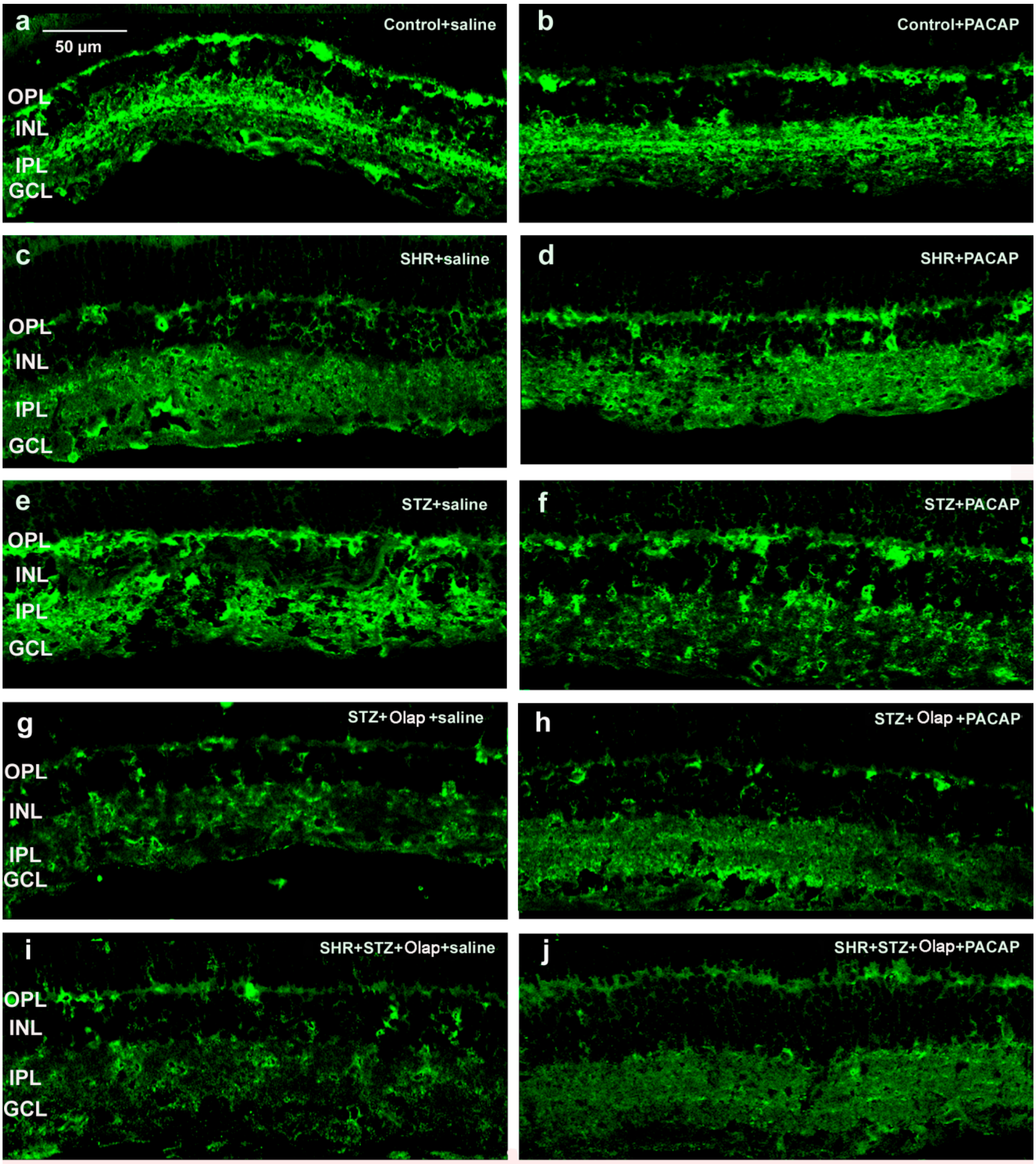


**Figure S4.** Calretinin immunoreactivity in retinal sections under different conditions: (**a**) untreated control; (**b**) PACAP treated control retina; (**c**) untreated hypertensive retina; (**d**) PACAP treated hypertensive retina, (**e**) untreated diabetic retina; (**f**) PACAP treated diabetic retina; (**g**) PARP inhibitor (Olap) treated hypertensive retina; (**h**) PACAP and PARP inhibitor (Olap) treated diabetic retina; (**i**) PARP inhibitor (Olap) treated hypertensive diabetic retina; (**j**) PACAP and PARP inhibitor (Olap) treated hypertensive diabetic retina. OPL- outer plexiform layer, INL- inner nuclear layer, IPL- inner plexiform layer, GCL-ganglion cell layer. Scale bar: 50µm.

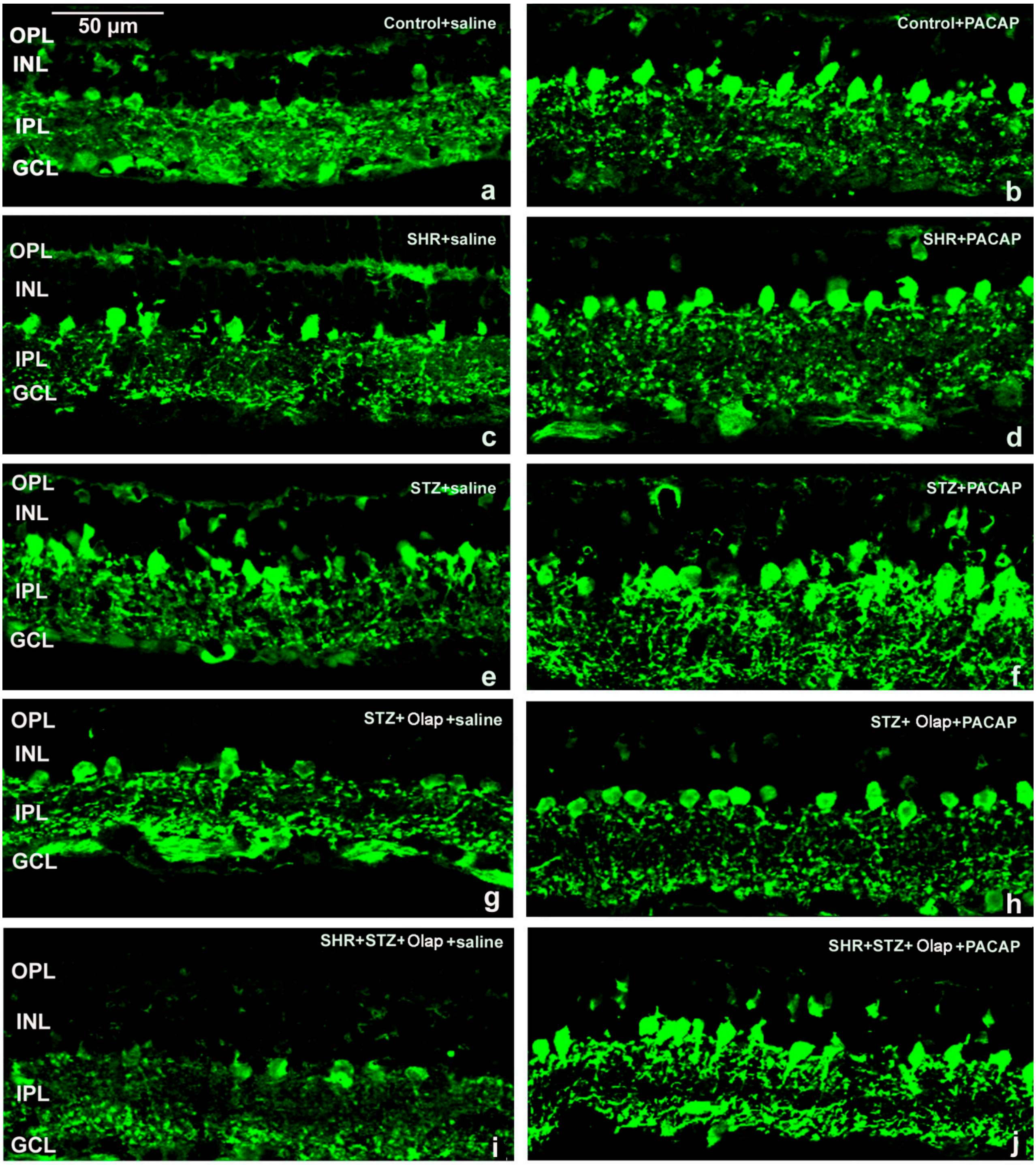


**Figure S5.** Parvalbumin immunoreactivity in retinal sections under different conditions: (**a**) untreated control; (**b**) PACAP treated control retina; (**c**) untreated hypertensive retina; (**d**) PACAP treated hypertensive retina, (**e**) untreated diabetic retina; (**f**) PACAP treated diabetic retina; (**g**) PARP inhibitor (Olap) treated diabetic retina; (**h**) PACAP and PARP inhibitor (Olap) treated diabetic retina; (**i**) PARP inhibitor (Olap) treated hypertensive diabetic retina; (**j**) PACAP and PARP inhibitor (Olap) treated hypertensive diabetic retina. OPL- outer plexiform layer, INL- inner nuclear layer, IPL- inner plexiform layer, GCL-ganglion cell layer. Scale bar: 50µm.

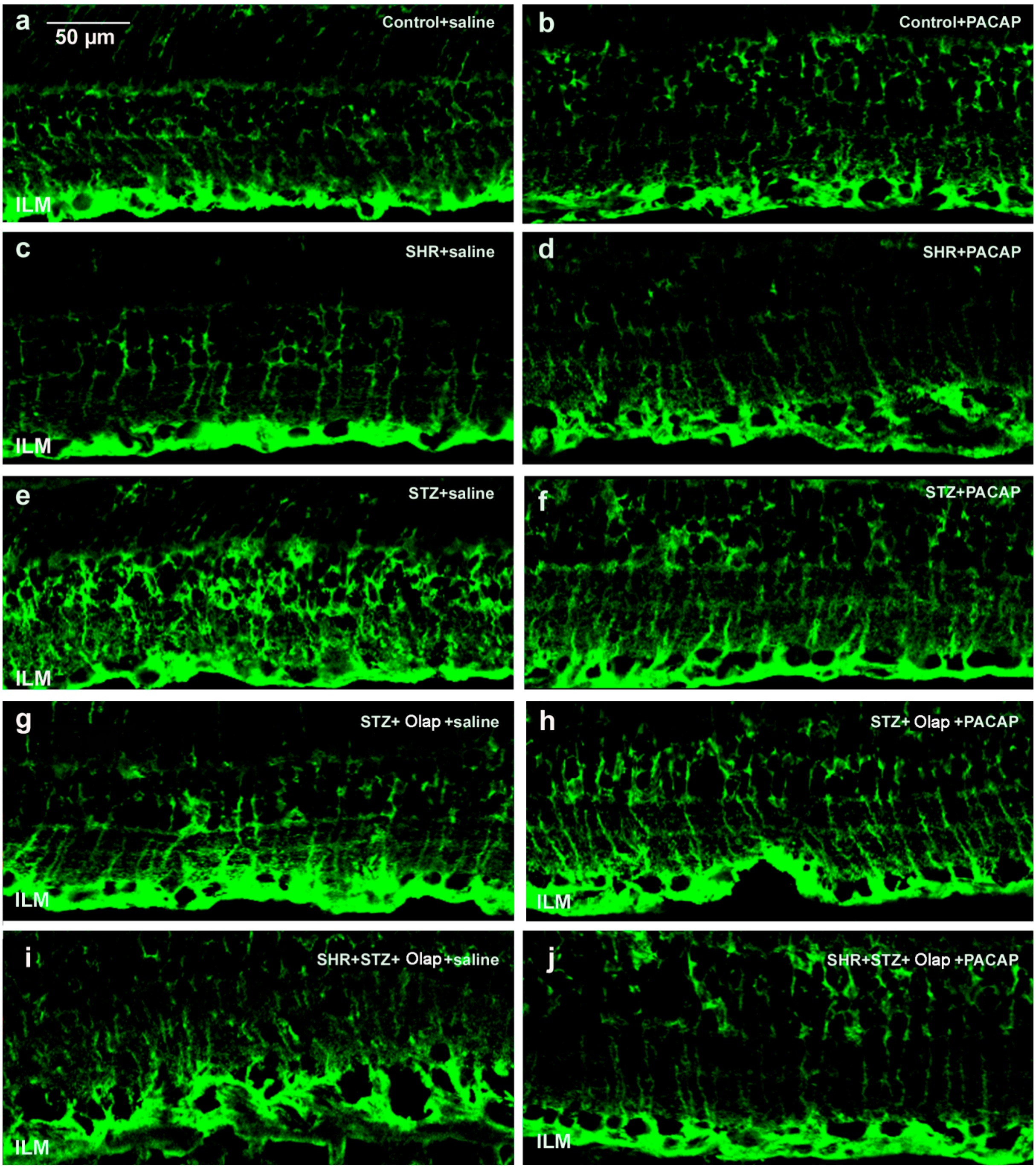


**Figure S6.** Representative retinal sections stained with GS in different conditions: (**a**) untreated control; (**b**) PACAP treated control; (**c**) untreated hypertensive; (**d**) PACAP treated hypertensive, (**e**) untreated diabetic; (**f**) PACAP treated diabetic; (**g**) PARP inhibitor (Olap) treated diabetic retina; (**h**) PACAP and PARP inhibitor (Olap) treated diabetic retina; (**i**) PARP inhibitor (Olap) treated hypertensive diabetic retina; (**j**) PACAP and PARP inhibitor (Olap) treated hypertensive diabetic retina. ILM- inner limiting membrane. Scale bar: 50µm.

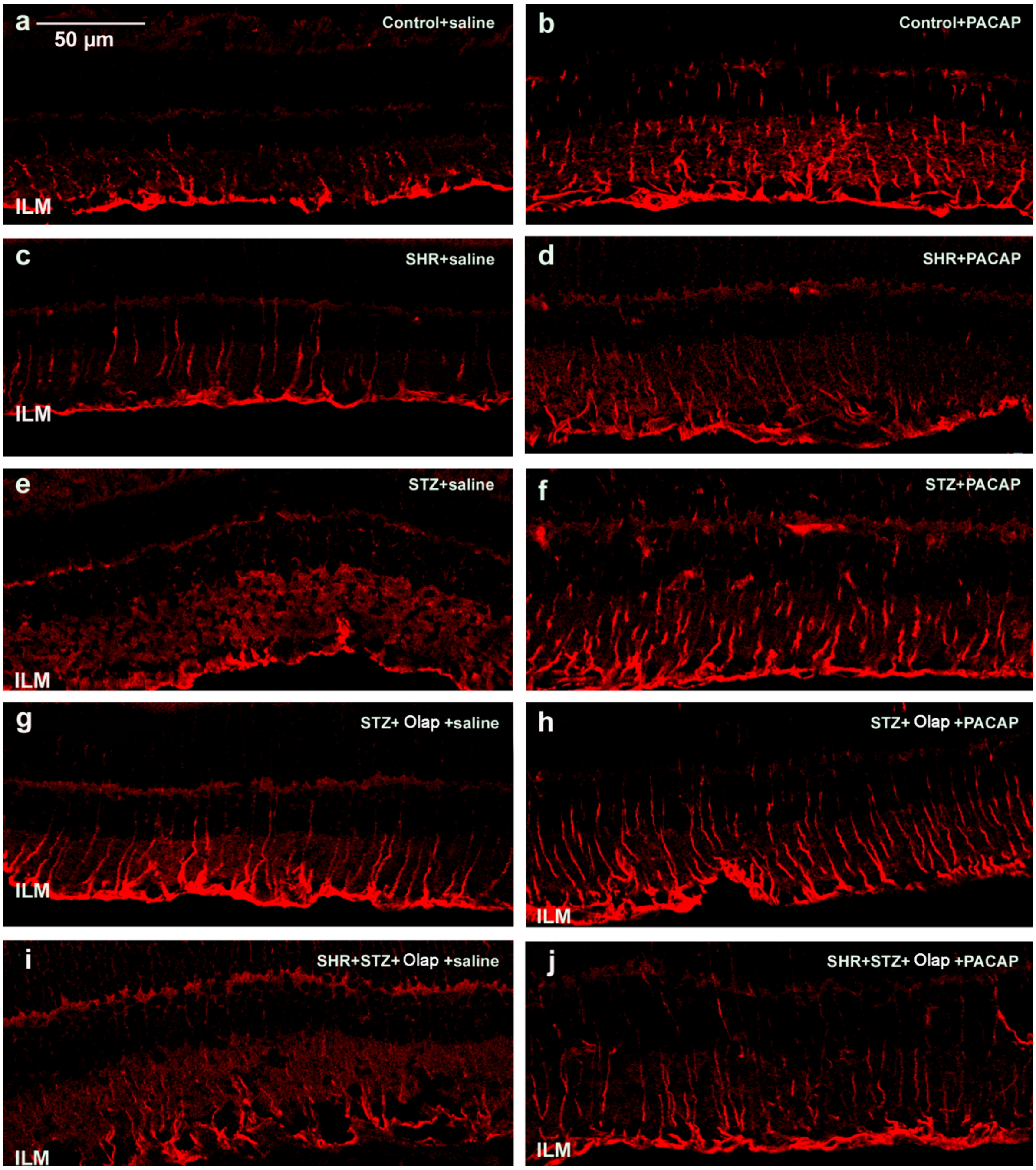


**Figure S7.** Representative retinal sections stained with Müller glia cell marker (GFAP) in different conditions: (**a**) untreated control retina; (**b**) PACAP treated control retina; (**c**) untreated hypertensive retina; (**d**) PACAP treated hypertensive retina, (**e**) untreated diabetic retina; (**f**) PACAP treated diabetic retina; (**g**) PARP inhibitor (Olap) treated diabetic retina; (**h**) PACAP and PARP inhibitor (Olap) treated diabetic retina; (**i**) PARP inhibitor (Olap) treated hypertensive diabetic retina; (**j**) PACAP and PARP inhibitor (Olap) hypertensive diabetic. ILM- inner limiting membrane. Scale bar: 50µm.

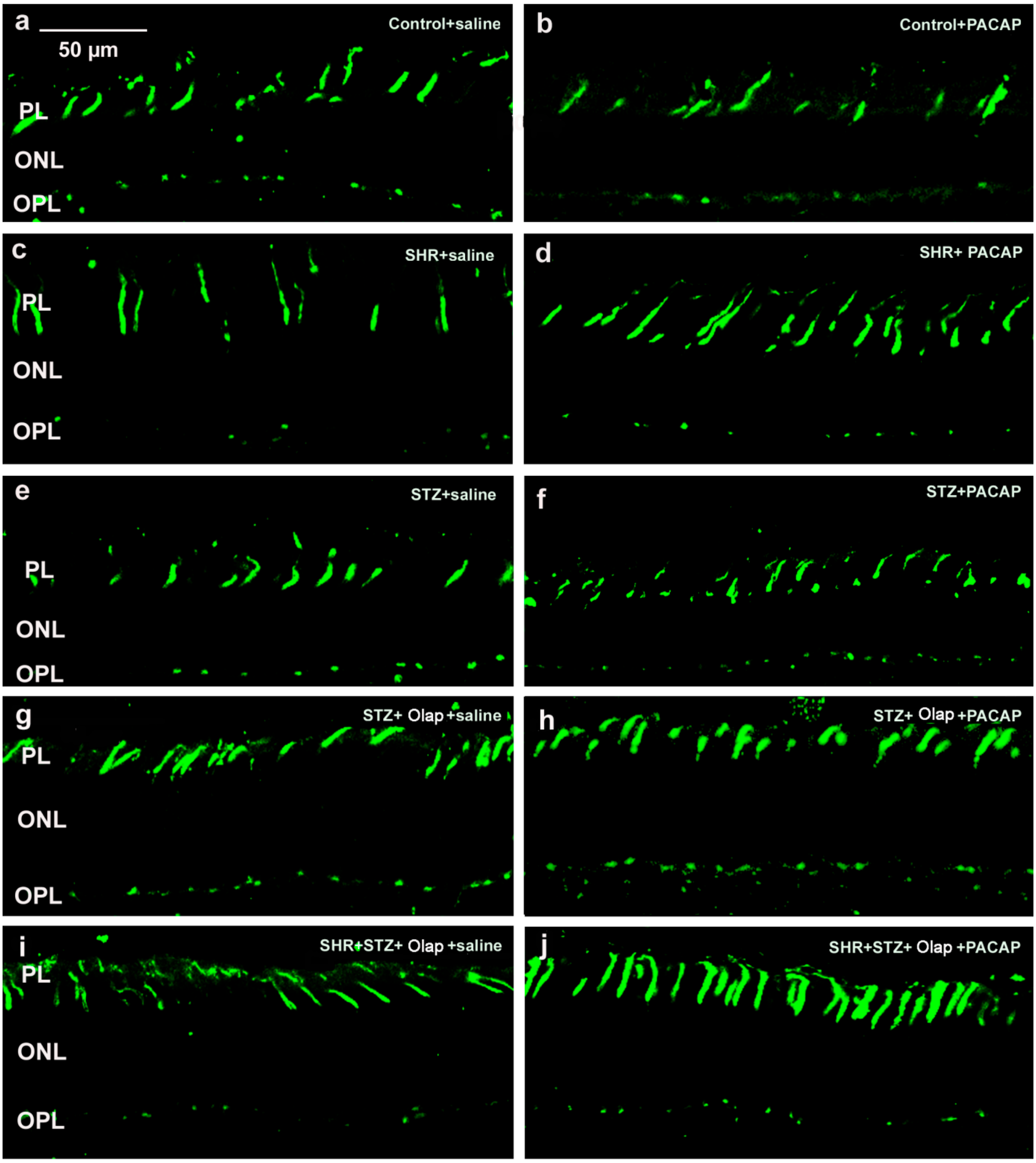


**Figure S8.** Representative retinal sections stained with cone terminal marker (PNA) in different conditions: (**a**) untreated control retina; (**b**) PACAP treated control retina; (**c**) untreated hypertensive retina; (**d**) PACAP treated hypertensive retina, (**e**) untreated diabetic retina; (**f**) PACAP treated diabetic retina; (**g**) PARP inhibitor (Olap) treated diabetic retina; (**h**) PACAP and PARP inhibitor (Olap) treated diabetic retina; (**i**) PARP inhibitor (Olap) treated hypertensive diabetic retina; (**j**) PACAP and PARP inhibitor (Olap) treated hypertensive diabetic retina. PL – photoreceptor layer; ONL – outer nuclear layer; OPL – outer plexiform layer. Scale bar: 50µm.

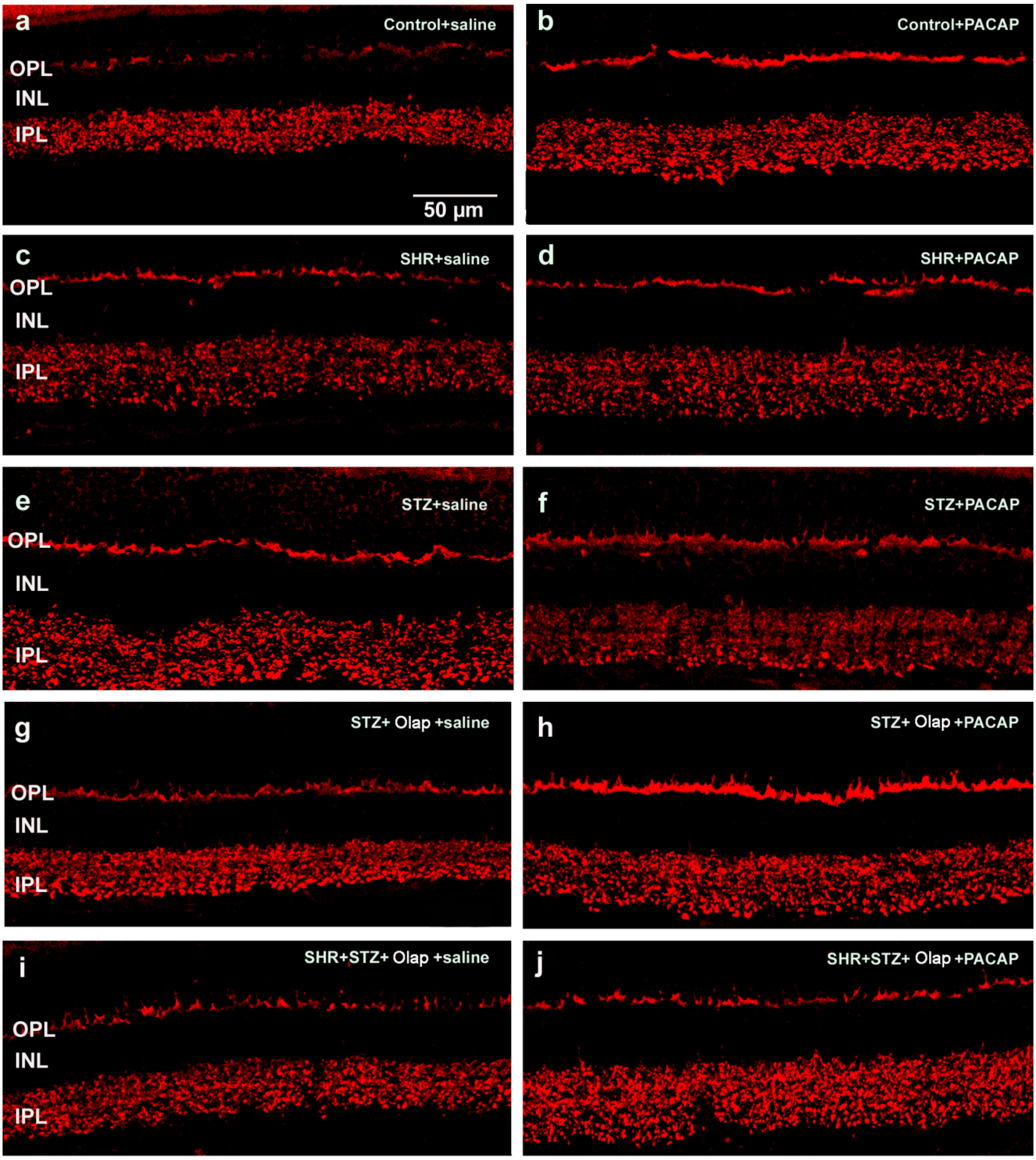


**Figure S9.** vGlut1 immunoreactivity in photoreceptor and bipolar cell terminals: (**a**) untreated control; (**b**) PACAP treated control retina; (**c**) untreated hypertensive retina; (**d**) PACAP treated hypertensive retina, (**e**) untreated diabetic retina; (**f**) PACAP treated diabetic retina; (**g**) PARP inhibitor (Olap) treated diabetic retina; (**h**) PACAP and PARP inhibitor (Olap) treated diabetic retina; (**i**) PARP inhibitor (Olap) treated hypertensive diabetic retina; (**j**) PACAP and PARP inhibitor (Olap) treated hypertensive diabetic retina OPL – outer plexiform layer, INL- inner nuclear layer, IPL- inner plexiform layer. Scale bar: 50µm.
